# Stimulant treatment for attention-deficit/hyperactivity disorder and risk of first and repeat criminal offending: a population data linkage cohort study

**DOI:** 10.23889/ijpds.v9i5.2714

**Published:** 2024-09-18

**Authors:** Timothy Nielsen, Ralph Nanan, Tony Butler, Natasha Nassar, Alison Poulton

**Affiliations:** 1 University of Sydney; 2 University of New South Wales

## Objective

To examine the effect of stimulant treatment on first and repeat criminal offending among children and young adults with attention deficit-hyperactivity disorder (ADHD).

## Approach

A population data linkage cohort study of individuals born in New South Wales (NSW), Australia in 1990-2005 and followed until May 2016. Individuals linked to stimulant treatment authorizations for ADHD in the NSW Ministry of Health controlled substance database were frequency matched (1:10) to controls without ADHD. Proven criminal offenses were linked from statewide court records. First and repeat offenses were examined separately using modified cox regression and Prentice, Williams, Peterson (PWP) models, respectively.

## Results

The cohort included 75,650 individuals with ADHD and 745,634 controls. A total of 59,658 individuals (7.3%) committed an offense and individuals with ADHD committed 3 times as many offenses. For all age and sex groups, the risk of a first offense was increased among individuals with ADHD but reduced by treatment (Males 10-17 years, untreated Hazard Ratio (HR) 2.02 95% confidence interval (CI) 1.95, 2.10, treated HR 1.52 95% CI 1.41, 1.62). The effect estimates were reduced for repeat offenses (Males 10-17 years, untreated HR 1.09 95% CI 1.05, 1.13, treated HR 0.97 95% CI 0.90, 1.04) with mixed results for the effect of treatment.

## Conclusions

Individuals with ADHD had increased risk of offending, but stimulant treatment reduced the risk of first offenses. This association and benefit of treatment were reduced among prior offenders.

## Implications

Adequate treatment may help keep young people with ADHD out of the criminal justice system.

